# The Effect of Serum 25-Hydroxyvitamin D on Serum Ferritin Concentrations: A Longitudinal Study of Participants of a Preventive Health Program

**DOI:** 10.3390/nu11030692

**Published:** 2019-03-23

**Authors:** Lalani L. Munasinghe, John P. Ekwaru, Silmara S. B. S. Mastroeni, Marco F. Mastroeni, Paul J. Veugelers

**Affiliations:** 1 School of Public Health, University of Alberta, Edmonton, AB T6G 2T4, Canada; munasing@ualberta.ca (L.L.M.); ekwaru@ualberta.ca (J.P.E.); 2 Department of Physical Education, University of Joinville Region, Joinville 89219-710, SC, Brazil; silmara.mastroeni@univille.br; 3 Post-graduation Program in Health and Environment, University of Joinville Region, Joinville 89219-710, SC, Brazil; marco.mastroeni@univille.br

**Keywords:** serum ferritin, vitamin D, inflammation, body weight status, cardiovascular disease, Canadians

## Abstract

Various studies have suggested a role of vitamin D in inflammation. However, its effect on ferritin, a biomarker of inflammation, has received relatively little attention. Therefore, we aimed to assess the association of serum 25-hydroxyvitamin D (25(OH)D) with serum ferritin (SF) concentrations, and to examine whether temporal increases in serum 25(OH)D concentrations are paralleled by a reduction in SF concentrations. Data from a community sample of Canadian adults who participated in a preventive health program (*n* = 6812) were analyzed. During the follow-up, serum 25(OH)D concentrations increased from 80.7 to 115.0 nmol/L whereas SF concentrations decreased from 122.0 to 92.0 µg/L (median follow-up time was 11.67 months). Cross-sectional analyses revealed that compared to participants with 25(OH)D concentrations of <50 nmol/L, those with 25(OH)D concentrations of 75 to <100, 100 to <125, and ≥125 nmol/L had SF concentrations that were 13.00, 23.15, and 27.59 µg/L lower respectively (*p* < 0.001). Compared to those without temporal improvements in 25(OH)D concentrations between baseline and follow-up, participants who improved their 25(OH)D concentrations with ≥50 nmol/L decreased their SF concentrations with 5.71 µg/L. For participants for whom the increase in 25(OH)D concentrations was less than 50 nmol/L, decreases in SF concentrations were less pronounced and not statistically significant. These observations suggest that despite strong associations between 25(OH)D and SF concentrations, interventions aiming to lower SF concentrations through sun-exposure and vitamin D supplementation should target substantial increases in 25(OH)D concentrations.

## 1. Introduction

Serum ferritin (SF) is the storage form of iron in the body. Low serum concentrations of ferritin in the body is indicative of a negative iron balance and anemia, i.e., iron deficiency anemia [1,2,3]. SF is also an acute-phase reactant that elevates as part of a systemic response to inflammation [3,4,5] and is therefore recognized as a marker of inflammation [3,6]. Chronic inflammation can cause anemia, i.e., “anemia of inflammation” or “anemia of chronic disease”, and high SF concentrations induced by chronic inflammation may obscure iron deficiency anemia [2]. Given the key role of chronic inflammation in the causation of multiple chronic diseases [7,8,9], maintaining proper SF concentrations is a recognized strategy in their prevention [10,11,12]. The identification of novel ways to prevent and control inflammation are essential to this strategy.

The prohormone nutrient vitamin D has a key function in bone metabolism and is increasingly recognized for its anti-inflammatory effect [13,14,15,16]. However, the potential influence of vitamin D on SF concentrations, as an inflammation marker, has received relatively little attention. Conclusions from existing and mostly cross-sectional studies are inconsistent [2,17,18,19,20,21,22,23]. Therefore, the objective of this study is to shed more light on the cross-sectional associations of serum 25-hydroxyvitamin D (25(OH)D) with SF concentrations, and, importantly, to examine whether temporal increases in serum 25(OH)D concentrations are paralleled by the reduction in the SF concentrations. The latter would suggest a potential to influence SF concentrations by increasing 25(OH)D concentrations.

## 2. Materials and Methods

### 2.1. Study Design and Participants

We analyzed data collected as part of a preventative and integrative health program offered to community volunteers by the Pure North S’Energy Foundation (PN), a not-for-profit charitable organization. The primary objective of the PN program is lifestyle counselling and disease prevention. At enrolment, participants complete a lifestyle questionnaire, give a medical history, and have biometric measurements taken (height, weight, waist circumference, blood pressure) and blood drawn for the assessment of several biomarkers including serum 25(OH)D and SF concentrations. The collected information is used by health professionals to provide informed lifestyle counselling that includes personalized recommendations on diet, physical activity, dietary and vitamin D supplements, sleep and stress management. Follow-up visits are scheduled annually for health assessments and lifestyle counseling. Further information about the program can be found on the PN website [24]. Data collected from October 2007 through to April 2014 with granted written informed consent for use of the information for research purposes were used for the current analyses. All data were anonymized by PN prior to it being transferred to the University of Alberta for data analyses. Ethical approval was obtained from the Human Research Ethics Board at the University of Alberta.

Participants aged ≥18 years at baseline and with complete information on serum 25(OH)D concentrations and SF concentrations, one at baseline and one at the first follow-up visit, were considered for the present study. Those known to have kidney failure, have been taking medication for kidney failure, with a glomerular filtration rate of less than 15 mL/min per 1.73 m^2^ or known to have cancer were excluded as those conditions themselves could cause elevated SF [25,26]. Participants with SF >1000 µg/L or C-reactive protein (CRP) concentration >10 mg/L were also excluded due to the possibility that such high concentrations were caused by an infection, an acute inflammation or an unknown condition rather than chronic inflammation [27]. The data for the present study included observations of 6812 participants, each with a baseline and a follow-up observation. The median time from baseline to follow-up was 11.67 months.

### 2.2. Serum Ferritin Concentrations

Immunoturbidimetric assay using the Beckman Coulter AU680^®^ chemistry analyzer (Mississauga, ON, Canada) measured SF concentrations. Immunoassay reagent, K-Assay^®^ Ferritin test kit (Cat. No. KAI-095), for use on the chemistry analyzer was obtained from Kamiya Biomedical (Seattle, WA, USA). The analytical measurable range was 6–1000 ng/L with coefficient of variance <5%. Temporal changes in SF concentrations were calculated by subtracting baseline concentrations from follow-up concentrations.

### 2.3. Serum 25(OH)D Concentrations

DiaSorin^®^ Liason chemiluminescent immunoassay with an inter-assay coefficient of variation of 11% was used to measure serum 25(OH)D concentrations. Serum 25(OH)D concentrations at baseline and follow-up were categorized as <50, 50 to <75, 75 to <100, 100 to <125 and ≥125 nmol/L. The change in 25(OH)D was calculated by subtracting the baseline concentration from the follow-up concentration. Changes were then categorized into “No Improvement”, “Increased by <25”, “Increased by 25 to <50”, and “Increased by ≥50 nmol/L”.

### 2.4. Potential Confounding Variables

Gender, age, body weight status, blood pressure, smoking, alcohol consumption, physical activity and ethnicity were considered as potential confounders. Body mass index (BMI) was calculated for each participant as weight in kg/height^2^ (kg/m^2^) and categorized as “Underweight” (BMI < 18.5 kg/m^2^), “Normal weight” (BMI = 18.5 to < 25 kg/m^2^), “Overweight” (BMI = 25 to < 30 kg/m2), and “Obesity” (BMI ≥ 30 kg/m^2^) [28]. Due to the small number of underweight participants, underweight and normal weight individuals were combined into a single group to achieve meaning group sizes in the regression analysis. Blood pressure status was defined as “Normal” if systolic and diastolic pressures were <120/80 mmHg, and as “Elevated” if they were ≥120/80 mmHg or if the participant was using an anti-hypertensive medication [29]. Smoking status was defined as “Never smoker”, “Past smoker”, and “Current smoker”. Alcohol consumption status was categorized as “Non-drinker” for those who reported drinking <2 glasses per week and “Drinker” for those who drank ≥ 2 glasses per week, and ethnicity as “White” and “Non-white”. Physical activity levels were determined by estimating the metabolic equivalent of task (MET) per week for each physical activity reported. MET values were then multiplied by the time participants reportedly spent performing those activities per week (MET × hours per week). The total for each week was categorized as “low (<10 MET hours/week)”, “moderate (10 to <20 MET hours/week)” and “high (≥20 MET hours/week)”. MET hours per week at baseline were subtracted from the MET hours per week at the follow-up visit to determine change in physical activity. Change in physical activity was categorized as “Negative change” if MET hours per week were greater at baseline than at the follow-up, “No change” if MET hours per week remained unchanged and “Positive change” if MET hours per week were greater at the follow-up than baseline. Participants were asked the type and amount of supplementation they were using. Based on their responses related to vitamin D and multivitamin supplementation, the amount of vitamin D was calculated. Missing observations for confounding variables were treated as separate covariate categories and grouped as “Missing”.

### 2.5. Statistical Analyses

Percentages, medians with interquartile range, and means with standard deviation at both baseline and the follow-up are presented as descriptive statistics. Multiple linear regression analyses with repeated measures were used to identify the cross-sectional association of serum 25(OH)D and SF concentrations at baseline and the follow-up. These associations were adjusted for the confounding potential of age, gender, body weight status, blood pressure status, smoking status, alcohol consumption, physical activity level, and ethnicity. Although the distribution of SF concentrations is skewed, the residuals of the regression model were not skewed: A log transformation of SF concentrations is therefore not needed. Multiple linear regression models without repeated measures were used to identify the relationship of temporal changes in 25(OH)D with temporal changes in SF concentrations. These analyses were adjusted for baseline SF concentration, baseline serum 25(OH)D concentration, baseline age, gender, baseline body weight status, baseline values for blood pressure status, smoking status, alcohol consumption, and physical activity, and ethnicity. Analyses were stratified by gender and body weight status because SF concentrations vary considerably across their subgroupings. The above-mentioned regression analyses were further adjusted for CRP concentrations to quantify the associations of 25(OH)D with SF independent of the effect of CRP. All statistical analyses were performed using Stata, version 15.0 (Stata Corp, College Station, TX, USA) with statistical significance at 0.05.

## 3. Results

Participant characteristics are shown in Table 1 and Table 2. Mean and median 25(OH)D concentrations at baseline, 87.2 and 80.7 nmol/L respectively, increased to 121.4 and 115.0 nmol/L respectively, at the follow-up. Mean and median SF concentrations at baseline, 160.6 µg/L and 122.0 µg/L respectively, decreased to 132.3 and 92.0 µg/L respectively, at the follow-up. The prevalence of elevated SF, 16.3% at baseline, also decreased to 11.5% at the follow-up (Table 1). Participants were 50.6 years of age, 30.0% was overweight and 26.4% obese (Table 2). At baseline, 46.9% of participants reported taking vitamin D-containing supplements with a median dose 3000 IU per day (Table 2), which increased to 75.8% with a median dose 7000 IU per day at the follow-up.

Table 3 depicts the cross-sectional associations of serum 25(OH)D and SF concentrations for the baseline and follow-up observations. Compared to participants with 25(OH)D concentrations of <50 nmol/L, those with 25(OH)D concentrations of ≥125 nmol/L had, on average, SF concentrations that were 27.59 µg/L lower. Participants with 25(OH)D concentrations of 75 to <100 nmol/L and of 100 to <125 nmol/L had statistically significant lower SF concentrations compared to those with 25(OH)D concentrations of <50 nmol/L. These differences appeared to be generally more pronounced among men and among those with excess body weight (Table 3). When further adjusted for the potential influence of CRP, the associations of serum 25(OH)D with SF concentrations appeared very similar in magnitude and statistical significance as those presented in Table 3.

Table 4 depicts the associations of temporal changes in 25(OH)D concentrations with temporal changes in SF concentrations. Compared to participants without temporal improvements in 25(OH)D concentrations between baseline and the follow-up, those who improved their 25(OH)D concentrations with ≥50 nmol/L had on average a decrease in their SF concentrations of 5.71 µg/L. For participants for whom their 25(OH)D concentrations increased with less than 50 nmol/L, the decrease in SF concentrations was less and not statistically significant. The only gender and weight status subgroups that depicted a statistically significant decrease in SF concentrations were obese participants, female and male participants combined; male participants; and obese male participants (Table 4). When further adjusted for the potential influence of CRP, the associations of temporal changes in 25(OH)D concentrations with temporal changes in SF concentrations appeared very similar in magnitude and statistical significance as those presented in Table 4. Statistically significant associations were observed only in those same subgroups where statistically significant associations were observed without the CRP adjustment (obese participants, female and male participants combined; male participants; and obese male participants).

## 4. Discussion

We compared serum 25(OH)D and SF concentrations both cross-sectionally and longitudinally. The cross-sectional comparisons revealed strong and mostly statistically significant inverse associations. In contrast, the longitudinal comparisons revealed modest associations for those participants who improved their 25(OH)D concentrations with ≥50 nmol/L compared to those who did not improve their 25(OH)D concentrations. These longitudinal comparisons showed the absence of statistically significant associations between 25(OH)D and SF concentrations for those participants who improved their 25(OH)D concentrations with <50 nmol/L, for participants with normal weight and participants who were overweight, and for participating women.

Cross-sectional studies reported high SF concentrations and a high prevalence of anemia based on low mean hemoglobin concentration among adults [23] and elderly [30] with vitamin D deficiency in the United States. Coutard et al. [31] in contrast, did not find this for hospitalized geriatric patients in France. Castro et al. [32] did not observe an association of vitamin D with SF concentrations among ambulatory patients with inflammatory bowel disease in Portugal, whereas Andiran et al. [33] did demonstrate such an association among children and adolescents who attended an outpatient clinic in Turkey. None of the above studies had considered potential confounders when examining the associations between vitamin D and SF, which complicates cross-study comparisons. A recent population-based cross-sectional study that considered important confounders reported inverse associations between serum 25(OH)D and SF among Korean men [18]. With respect to Korean women, they reported a positive association for pre-menopausal women and an absence of an association for post-menopausal women [18], following an earlier report of iron deficiency anemia and anemia of inflammation for both pre- and post-menopausal women of low vitamin D status [34]. In the present study, the cross-sectional comparisons had revealed an inverse association for both women and men, although the magnitude of the associations was somewhat more pronounced for men compared to women. Another recent population-based cross-sectional study that considered various confounders reported inverse associations for normal weight Canadians but not for overweight and obese Canadians [35]. This seems to contrast the cross-sectional observations of the present study where the associations among obese participants were more pronounced. We note important differences across the studies: The Korean study [34] and the Canadian study [35] were population-based whereas the present study included volunteer participants of a preventive health program with the majority of participants reportedly using vitamin D supplements in a country where the prevalence of iron deficiency anemia is low because of mandatory fortification of wheat flour [36].

The potential of vitamin D to lower SF concentrations has received little attention in intervention studies. The single randomized controlled trial in this area demonstrated that supplementation with 400 to 1000 IU of vitamin D per day for a period of 16 weeks increased serum 25(OH)D concentration from 28.7 nmol/L to 48.8 nmol/L while SF concentrations remained unchanged [20]. The present study evaluated a preventive health program where participants used higher doses (at the follow-up, median doses were 7000 IU per day) which achieved serum 25(OH)D concentrations to increase, on average, from 87.2 nmol/L to 121.4 nmol/L. Only in the subgroup with increases in excess of 50 nmol/L, did we observe a statistically significant reduction in SF concentrations. Subgroups with smaller increases did not show a decrease in SF concentrations. Because in the above mentioned randomized controlled trial the increase in 25(OH)D concentrations was on average 20.1 nmol/L (48.8–28.7) [20], both studies seem consistent in their findings that increases in 25(OH)D concentrations of less than 50 nmol/L do not affect SF concentrations.

Current vitamin D recommendations by the Institute of Medicine are to achieve 25(OH)D concentrations of 50 nmol/L as this level ensures good bone health [37]. Other institutions, include the Endocrine Society [38], the National Osteoporosis Society [39] Osteoporosis Canada [40], the Multiple Sclerosis Society of Canada [41], and the American Geriatrics Society [42], recommend higher serum 25(OH)D concentrations (≥75 nmol/L) with the aim to achieve various other health benefits. An increase of 25 nmol/L (i.e., from the recommendation of 50 nmol/L to the recommendation of 75 nmol/L) does not seem enough to affect SF concentrations as the present study revealed that only participants who improved their 25(OH)D concentrations with ≥ 50 nmol/L showed reductions in SF concentrations. These associations appeared largely independent of the influence of CRP. Another investigation of the potential anti-inflammatory role of vitamin D had suggested that obese subjects reduced the risk of having elevated CRP serum concentrations by increasing their serum 25(OH)D concentrations [15]. This reduction in the risk of elevated CRP concentrations among obese subjects occurred in response to not only increases in 25(OH)D concentrations of ≥ 50 nmol/L, but also for smaller changes [15]. As both inflammation markers, SF and CRP, also predict adverse cardiovascular events, we recommend intervention studies that achieve high 25(OH)D concentrations to establish their combined benefits in terms of preventing cardiovascular disease among obese subjects.

Strengths of the present study include its longitudinal design, large sample size, the availability of information on various confounders and the wide range of serum 25(OH)D concentrations. We acknowledge that this study was conducted among volunteer participants of a preventive health program who are not representatives of the general population. The preventive health program not only encourages supplementation with vitamin D, but rather healthy lifestyles in general. The latter may have affected the findings of the present study. Caution is therefore warranted in the interpretation and generalization of the present findings.

## 5. Conclusions

The present study suggests that despite strong cross-sectional associations between 25(OH)D and SF concentrations, interventions that aim to lower SF concentrations through sun-exposure and vitamin D supplementation should target substantial increases in 25(OH)D concentrations. We recommend such intervention studies to establish the combined anti-inflammatory and cardiovascular benefits.

## Figures and Tables

**Table 1:** Serum 25(OH)D and serum ferritin levels at baseline and at the follow-up of 6812 study participants.

Characteristic	At Baseline	At Follow-Up
**Serum 25(OH)D, nmol/L**		
Mean (SD)	87.2 (41.6)	121.4 (48.9)
Median (IQR)	80.7 (59.5–106.0)	115.0 (88.0–148.0)
**Serum ferritin, µg/L**		
Mean (SD)	160.6 (139.5)	132.3 (126.0)
Median (IQR)	122.0 (64.0–212.0)	92.0 (47.0–174.0)
**Elevated serum ferritin, %**		
No	83.7	88.5
Yes (≥300 ng/mL for males and ≥200 ng/mL for females)	16.3	11.5

Abbreviations: 25(OH)D—25 hydroxy vitamin D; SD—Standard Deviation; IQR—Inter Quartile Range.

**Table 2:** Baseline characteristics of 6812 study participants.

Characteristics	
**Age, years**	
Mean (SD)	50.6 (15.2)
**Gender (%)**	
Female	51.8
Male	48.2
**Body weight status, %**	
Under weight	1.2
Normal weight	34.4
Overweight	38.0
Obesity	26.4
**Blood pressure, %**	
Normal (<120/80 mmHg)	31.1
Elevated (≥120/80 mmHg or anti-hypertensive medication use)	65.4
Missing	3.4
**Smoking status, %**	
Never smoker	39.7
Ex-smoker	21.8
Current smoker	9.3
Missing	29.2
**Alcohol consumption status, %**	
Non-drinker	31.1
Drinker	36.4
Missing	32.4
**Physical activity level, %**	
Low	28.6
Moderate	21.5
High	20.9
Missing	28.9
**Ethnicity**	
White	63.4
Non-white	36.6
**Use of vitamin D-containing supplements, %**	
No	35.3
Yes	46.9
Missing	17.8
**Vitamin D dose of the supplements, Median (IQR) IU/day**	3000 (2000–5000)

Abbreviations: SD—Standard Deviation; IQR—Inter Quartile Range.

**Table 3:** Cross-sectional associations of serum 25(OH)D and serum ferritin concentrations at baseline and follow-up.

	Number of Observations	All Participants		Under/Normal Body Weight		Overweight and Not Obese		Obesity	
		β (95% CI)	*p*	β (95% CI)	*p*	β (95% CI)	*p*	β (95% CI)	*p*
**All observations (*n* = 13624)**									
25(OH)D, nmol/L									
<50	1289	ref		ref		ref		ref	
50 to <75	2729	−3.36 (−9.60, 2.88)	0.291	1.75 (−8.76, 12.26)	0.744	0.97 (−10.53, 12.47)	0.869	−6.77 (−18.00, 4.45)	0.237
75 to <100	3193	−13.00 (−19.34, −6.65)	<**0.001**	−1.56 (−11.82, 8.70)	0.765	−11.90 (−23.47, −0.33)	**0.044**	−15.27 (−27.43, −3.10)	**0.014**
100 to <125	2574	−23.15 (−29.91, −16.39)	<**0.001**	−8.46 (−19.05, 2.14)	0.118	−17.68 (−29.95, −5.40)	**0.005**	−40.19 (−53.67, −26.70)	<**0.001**
≥125	3839	−27.59 (−34.68, −20.51)	<**0.001**	−12.39 (−23.25, −1.53)	**0.025**	−27.60 (−40.51, −14.68)	<**0.001**	−33.02 (−47.51, −18.53)	<**0.001**
**Among females (*n* = 7062)**									
25(OH)D, nmol/L									
<50	412	ref		ref		ref		ref	
50 to <75	1207	−6.51 (−12.85, −0.17)	**0.044**	2.43 (−8.21, 13.07)	0.654	−6.54 (−19.26, 6.17)	0.313	−11.63 (−23.00, −0.26)	**0.045**
75 to <100	1722	−13.67 (−20.02, −7.32)	<**0.001**	1.37 (−8.95, 11.69)	0.795	−14.98 (−27.55, −2.42)	**0.019**	−23.35 (−35.66, −11.05)	<**0.001**
100 to <125	1484	−14.38 (−21.03, −7.73)	<**0.001**	−0.57 (−11.11, 9.98)	0.916	−13.94 (−26.98, −0.90)	**0.036**	−26.85 (−40.34, −13.36)	<**0.001**
≥125	2237	−20.65 (−27.56, −13.75)	<**0.001**	−4.75 (−15.35, 5.85)	0.38	−22.71 (−36.52, −8.90)	**0.001**	−31.26 (−45.87, −16.65)	<**0.011**
**Among males (*n* = 6562)**									
25(OH)D, nmol/L									
<50	877	ref		ref		ref		ref	
50 to <75	1522	1.71 (−8.92, 12.34)	0.753	7.46 (−14.67, 29.59)	0.509	5.81 (−11.11, 22.74)	0.501	−2.32 (−20.89, 16.25)	0.807
75 to <100	1471	−8.97 (−19.98, 2.05)	0.111	2.13 (−19.82, 24.09)	0.849	−8.96 (−26.24, 8.32)	0.31	−11.64 (−31.86, 8.58)	0.259
100 to <125	1090	−32.22 (−44.20, −20.25)	<**0.001**	−16.49 (−39.88, 6.89)	0.167	−21.35 (−40.06, −2.64)	**0.025**	−52.99 (−75.41, −30.58)	<**0.001**
≥125	1602	−34.75 (−47.45, −22.04)	<**0.001**	−22.91 (−48.07, 2.24)	0.074	−31.27 (−50.82, −11.73)	**0.002**	−32.94 (−56.78, −9.11)	**0.007**

*p*-values from multiple linear regression model were adjusted for gender (except the gender stratified analyses), age, body weight status (except the analyses stratified by body weight status), blood pressure status, smoking status, alcohol status, physical activity level and ethnicity.

**Table 4:** Associations of temporal changes in serum 25(OH)D concentrations with coinciding changes in serum ferritin concentrations by gender and body weight status.

	Number of Participants	All Participants		Under/Normal Body Weight		Overweight and Not Obese		Obesity	
		β (95% CI)	*p*	β (95% CI)	*p*	β (95% CI)	*p*	β (95% CI)	*p*
**All observations (*n* = 6812)**									
Change in 25(OH)D, nmol/L									
No improvement	1390	ref		ref		ref		ref	
Increase by <25	1667	1.85 (−3.04, 6.74)	0.458	0.43 (−5.71, 6.58)	0.889	3.62 (−4.58, 11.82)	0.386	−1.90 (−13.30, 9.49)	0.743
Increase by 25 to <50	1562	−2.23 (−7.35, 2.89)	0.394	−1.96 (−8.41, 4.49)	0.551	−0.88 (−9.35, 7.60)	0.839	−5.60 (−17.62, 6.41)	0.360
Increase by ≥50	2193	−5.71 (−10.61, −0.82)	**0.022**	−3.16 (−9.22, 2.90)	0.307	−5.84 (−14.01, 2.33)	0.161	−12.62 (−24.16, −1.08)	**0.032**
**Among females (*n* = 3531)**									
Change in 25(OH)D, nmol/L									
No improvement	745	ref		ref		ref		ref	
Increase by <25	864	0.003 (−3.70, 3.70)	0.999	−0.59 (−5.26, 4.08)	0.804	−3.47 (−10.90, 3.94)	0.358	4.24 (−4.60, 13.09)	0.347
Increase by 25 to <50	824	−1.84 (−5.73, 2.03)	0.351	0.78 (−4.16, 5.73)	0.756	−6.56 (−14.24, 1.13)	0.095	0.67 (−8.48, 9.82)	0.886
Increase by ≥50	1098	−0.94 (−4.69, 2.80)	0.622	−0.17 (−4.75, 4.41)	0.941	−5.62 (−13.17, 1.93)	0.144	4.20 (−5.06, 13.46)	0.373
**Among males (*n* = 3281)**									
Change in 25(OH)D, nmol/L									
No improvement	645	ref		ref		ref		ref	
Increase by <25	803	2.96 (−6.05, 11.96)	0.520	3.68 (−12.52, 19.88)	0.656	7.89 (−4.77, 20.56)	0.222	−4.60 (−23.30, 14.10)	0.629
Increase by 25 to <50	738	−3.19 (−12.62, 6.25)	0.508	−3.59 (−20.34, 13.16)	0.674	0.40 (−12.67, 13.47)	0.952	−9.06 (−29.12, 10.99)	0.375
Increase by ≥50	1095	−9.75 (−18.69, −0.82)	**0.032**	−7.87 (−24.17, 8.42)	0.343	−5.35 (−17.75, 7.04)	0.397	−19.77 (−38.22, −1.31)	**0.036**

*p*-values from multiple linear regression model were adjusted for baseline serum ferritin, baseline serum 25(OH)D concentration, baseline age, baseline body weight status (except the analyses stratified by body weight status), baseline blood pressure status, baseline smoking status, baseline alcohol status, baseline physical activity level, ethnicity and gender (except the gender stratified analyses).
